# Cross- & multi-lingual medication detection: a transformer-based analysis

**DOI:** 10.1186/s12911-025-03179-1

**Published:** 2025-10-02

**Authors:** Lisa Raithel, Johann Frei, Philippe Thomas, Roland Roller, Pierre Zweigenbaum, Sebastian Möller, Frank Kramer

**Affiliations:** 1 Universität Augsburg, Alter Postweg 101, Augsburg, 86159 Germany; 2 Quality & Usability Lab, Technische Universität Berlin, Ernst-Reuter Platz 7, Berlin, 10587 Germany; 3 BIFOLD – Berlin Institute for the Foundations of Learning and Data, Ernst-Reuter Platz 7, Berlin, 10587 Germany; 4 DFKI GmbH, Alt-Moabit 91c, Berlin, 10559 Germany; 5 Laboratoire interdisciplinaire des sciences du numérique (LISN), Université Paris-Saclay, Orsay, 91405 France

**Keywords:** Natural language processing, Information extraction, Medication detection, Multi-linguality

## Abstract

Extracting specific information, such as medication mentions, from large unstructured medical texts can be challenging, especially when no annotated corpus exists in the target language for training. To overcome this, leveraging existing machine learning models and datasets is essential, and since most pre-trained resources are in English, adopting multilingual approaches can help transferring between languages. In this work, we investigate the usage of a multi-lingual transformer model in a multi-lingual and cross-lingual setting to extract drug names from medical texts using named entity recognition in four European languages: German, English, French, and Spanish. We report the scores obtained by cross-lingual transfer with several published datasets after fine-tuning a multi-lingual model, aiming to create empirical evidence on how the transfer of “medical” knowledge between languages can be expected to benefit various language pairs. We further perform a qualitative error analysis and find that the performance on all languages achieves competitive levels. Conversely, erroneous prediction artifacts are introduced by annotation inconsistencies, differences in annotation guidelines and vague entity labels in general.

**Supplementary Information:**

The online version contains supplementary material available at 10.1186/s12911-025-03179-1.

## Introduction

Recent developments in Natural Language Processing (NLP), like the publication and democratization of transformer models [1], have allowed tremendous improvements also in biomedical text processing [2, 3]. For instance, in the n2c2 2022 medication detection challenge [4]^1^ (Track 1, Subtask 1), conducted as a named entity recognition (NER) task, the 13 highest ranking participants relied on transformer architectures and all achieved an *F*_1_ score above 96% (relaxed match) in detecting medication mentions in English electronic health records. However, while automatically processing medical text bears many different hurdles, such as data access or limited computation power in hospital infrastructures, the fact that most resources only exist for English is an additional challenge for many researchers around the globe.

Especially in classical, mono-lingual-based approaches, a low resource setting, or even a lack of matching annotated corpora for a certain target language is not uncommon. Our work investigates the use of multilingual medication detection for German (de), French (fr), and Spanish (es), in addition to English (en) in the medical context. The core question that we address concerns the extent to which existing data and multi-lingual models can improve the situation in non-English medical NLP settings. In practical terms: Which performance can we expect if we rely on an annotated corpus from a source language as training data and apply the subsequently trained model on a target language?

Finding evidence-based answers to this research question has clear practical implications. (i) Multi-lingual models facilitate common interoperability across multiple languages and, for instance, can be more effective in actual deployments since a multi-lingual base model does not need to be swapped if the input language is changed, in contrast to mono-lingual base models. (ii) Utilizing multi-lingual models allows the composition of data sources from multiple languages and therefore, it mitigates data scarcity in the medical context. (iii) In cases of such dataset scarcities in certain languages, cross-lingual approaches might be able to bridge the knowledge from a dataset in a source language to be applied in a target language.

Our experimental study design focuses on the cross-lingual capabilities of masked language models, which offer a suitable architecture for NER tasks. While much attention has been dedicated to large language models (LLMs) recently, these causal language models substantially differ in conceptual and practical terms, rendering a fair and exhaustive comparison of LLMs and BERT models challenging. Therefore, the scope of this study does not include LLM-driven medication detection methodologies.

To this end, we employ a multi-lingual transformer model for medication detection. It facilitates our evaluation setup since drug-related labeled data from different languages and different medical corpora are available and hence, multiple cross-lingual transfer settings can be conducted.

We report precision, recall, and *F*_1_ scores for different dataset configurations as part of the evaluation. To expand on the scores, we additionally contribute a comprehensive error analysis of the resulting predictions based on sample-wise observations to find artifacts and error patterns that are not well-captured by the plain scores. Within this scope, we try to categorize common errors across and within languages and corpora and set them into context. A summary of general observations made on the predicted entities concludes the analysis.

## Related work

In recent years, transformer-based approaches have achieved strong results in common language modeling tasks [1]. While most neural nets such as BERT [5] are frequently optimized for monolingual settings like English data, attempts to model multiple languages by a single network jointly have been described and successfully demonstrated with mBERT [5], XLM [6] or XLM-RoBERTa [7].

The properties and capabilities of multi-lingual models have been analyzed in several works. For instance, Pires et al. [8] investigated the mBERT model regarding multi-lingual transfer and reported indications that mBERT learns implicit multi-lingual representations, yet strong transfer capabilities are rather limited to “topologically similar” language pairs. Similarly, Wu and Dredze [9] evaluated the zero-shot performance and behavior of mBERT across several NLP tasks with positive results, yet they regard cross-lingual transfer in low-resource languages as future work. Chai et al. [10] discussed several linguistic factors for multi-lingual transfer on mBERT and XLM-R and identified word composition as a major contributor to multi-lingual understanding while constituent word ordering and word co-occurrences are of less importance. The cross-lingual transfer capabilities of models such as XLM-R has been also investigated by Al-Duwais et al. [11], including the transfer between English and Arabic. In their study about non-medical NER, the results show that the effectiveness of cross-lingual transfer highly depends on the target language.

Since multi-lingual networks can process data across different languages through shared weights without explicit parallel corpora [8], the idea of utilizing these models for performance improvement has been applied in countless contexts beyond biomedical or medical domains. In particular, low-resource contexts are of common interest due to the limited access to labeled data in the low-resource language and domain. Xie et al. [12] approached this issue by creating a shared embedding space for word translation and NER in a low-resource context instead of using jointly inherently multi-lingual networks like XLM-R or mBERT to model semantic similarities in an end-to-end fashion. For instance, using recent multi-lingual transformer models, Chen et al. [13] have applied XLM-R and cross-lingual pre-training to improve NER tasks on low-resource Uyghur and Hungarian datasets.

In the medical and biomedical domain, Catelli et al. [14] have fine-tuned a pre-trained mBERT model on Italian and English data to obtain NER items in Italian clinical texts for de-identification, yet the NER classes have no particular medical relationship. Concerning medical NER entities, Ding et al. [15] showed that bi-lingual models can improve NER performance by additionally pre-training on parallel corpora in English and Chinese. Their approach uses aligned ICD-11  [16] data for parallel text corpora, the MIMIC-III dataset [17] and internal Chinese data, and is built upon the XLM [6] model. Purwitasari et al. [18] trained mBERT and XLM-R in monolingual, zero-shot and joint multi-lingual training setups to evaluate the language transfer for English and Indonesian biomedical NER. The authors report superior performance of XLM-R compared to mBERT and no notable difference between the monolingual model and their multilingual approach. Contextualized embeddings from XLM-R models can also be applied without fine-tuning in a zero-shot fashion using embedding-based similarity search to detect medical entities in multi-lingual settings as demonstrated by Schwarz et al. [19]. For medical text classification, baseline experiments on cross- and multi-lingual transfer e.g. in the context of adverse drug reactions (ADR) [20] were conducted. The reported results on text classification using XLM-R show imperfect performance score for the transfer from English to German, which may be impacted by several factors such as the challenging ADR task, the varying data quality of social media forums, and class imbalances. Regarding the cross- and multi-lingual transfer from Chinese to English on medical NER, rather poor results (about 40% F1 score) have been reported [21] in their baseline systems. Zanoli et al. [22] report baseline experiments on the E3C corpus, focusing on disorder entities with mention- and concept-level UMLS annotations. Their results cover monolingual, multilingual, and cross-lingual training across English, French, Italian, Spanish, and Basque, though without analysis of specific language-to-language transfer. The work does not include German data or medication entities but provides useful baselines for disorder-focused biomedical NER.

Rather than only relying on cross-lingual transfer, the re-use of datasets from other languages through translation and annotation projection is considered. Concerning the question to which degree translation and annotation projection-based language shifts can outperform cross-lingual training, Gaschi et al. [23] investigated the unidirectional transfer from English to German and from English to French using the n2c2 2018 dataset (ADE, Track 2). Since their underlying evaluation corpora follow the identical annotation guidelines, the reported F1 score ranges between 72% (German) and 79% (French) using XLM-R base model for cross-lingual transfer. Similarly, Schafer et al. [24] is conceptually related, whereas both an annotation projection attempt is compared to an English-to-German cross-lingual transfer approach. They report a lower F1 score of 69% for the XLM-R base model on medication detection, which might also be influenced by the fact that their corpora do not share common annotation guidelines.

Large Language Models (LLMs) may also be used for medical-related tasks such as drug detection, taking advantage of their large training corpus and model size in multi-lingual tasks. However, LLM-based NER using few-short learning is reported to still perform inferior to masked language models [25, 26].

## Material and methods

Throughout this work, we refer to *cross-lingual* when all languages of the training set differ from the languages of the test set, and similarly, we consider the term *multi-lingual* if datasets from multiple languages are used jointly for training. In order to investigate the cross- and multi-lingual transferability for medication mention detection, we fine-tune a multi-lingual transformer model on several dataset compositions from different languages. Therefore, we harmonize all individual drug-related label classes by mapping all semantically similar label classes of each dataset to one harmonized label class which is subsequently used for all datasets.

If possible, we use not only one dataset per language but several to avoid overfitting on a particular kind of data or annotation style and to increase the model’s robustness on different text styles. Based on preliminary experiments, we select XLM-RoBERTa [7] as our transformer model, henceforth abbreviated as XLM-R.

Our approach investigates three perspectives. First, we fine-tune XLM-R mono-lingually as a mono-lingual reference. Second, we measure the performance in the joint multi-lingual setting by fine-tuning across all datasets from different languages. Third, we evaluate different combinations of training and test sets that allow us to quantify cross-lingual strengths and weaknesses for different languages. To provide further insights to our quantitative analysis, we also report and discuss observed artifacts and patterns in the qualitative analysis counterpart.

### Data overview

The presented corpora are selected because of the languages they represent and their respective annotations of medical entities. In particular, we are interested in medication names or other closely related types, such as substances. However, available medical datasets in languages other than English are limited, so we choose two Germanic (English and German) and two Romance (French and Spanish) languages and collect the corpora to which we were permitted access.

In particular, for the data, we consider medication names (and chemicals) used in medical texts, e.g., patient records. Usually, there is only one label per dataset dedicated to the desired expressions; sometimes, however, these labels cover a broader scope than only drug names, which is an inherent limitation when dealing with diverse datasets. As pointed out earlier, we harmonize all corpus-specific label classes we are interested in into one common label class. Such label classes are highlighted in **bold** in the subsequent descriptions of the datasets (Sections “German Datasets” and “Spanish Datasets”). The statistics of the available corpora and their selected drug-related labels can be found in Table 1. Due to pre-processing steps involving unicode normalization and span-corrections, certain text samples were removed to avoid issues with corrupted label spans after the unicode normalization.

**Table 1:** The dataset statistics. The data was tokenized using SpaCy [27]. Only labels of drug-related entity classes are counted. Number of tokens refers to the entire dataset

Dataset		# Tokens (overall)	# Labels (drug-related)
de	BRONCO150	83,551	1,630
	GERNERMED	21,678	1,450
	GGPONC 2.0	2,005,183	23,671
	Ex4CDS 2.0	4,356	98
en	CMED	472,114	8,993
fr	Quaero	79,706	3,537
	DEFT	284,111	1,337
es	PharmaCoNER	406,316	4,448
	CT-EBM-SP	355,443	9,224
Total		3,712,458	54,388

#### German datasets

##### BRONCO150 [28]

The Berlin-Tübingen Oncology Corpus^2^ contains 150 discharge summaries of cancer patients who received treatment at either Charité Berlin or Universitätsklinikum Tübingen. The summaries were manually anonymized, split into sentences, and scrambled to avoid the possibility of tracing back discharge reports to individuals. The sentences in this corpus are annotated with three entity labels (“diagnosis”, “treatment” and “**medication**”) and normalized to the terminologies ATC  [29] (medication), ICD-10  [30] (diagnosis), and OPS  [31] (treatment). Only complete tokens were annotated, even if only a sub-token was part of a medical entity. The authors define a medication as “a pharmaceutical substance or a drug that can be related to the Anatomical Therapeutic Chemical Classification System (ATC 3)” [28].

##### GERNERMED [32]

This corpus^4^ originates from the n2c2 2018 ADE dataset [33] which is an annotated English dataset that covers several medical entities such as “**Drug**”, “Dosage”, “Strength” etc. The German data samples are obtained through an automatic machine translation, while annotation information is transferred into German through word alignment estimation. Therefore, it is not a gold standard dataset. In this work, we use a refined dataset iteration which is available on request. According to the respective n2c2 annotation guideline, the drug entity should include all kinds of drugs except for “illicit” drugs and alcohol.

##### GGPONC v2.0 [34]

This datatset^5^ is, according to the authors, the first data collection based on clinical practice guidelines in German. GGPONC is a collection of curated scientific text documents, i.e., clinical guidelines that include, for example, instructions for the treatment of breast or lung cancer. It does not contain any personal data and thus is freely accessible. The entities labeled in this corpus belong to the category “Finding”, “**Substance**” or “Procedure”. The “Substance” label includes “general substances, the chemical constituents of pharmaceutical/biological products, body substances, dietary substances and diagnostic substances (…)”.^6^ The GGPONC 2.0 corpus provides both a short-span and a long-span annotation layer, determining whether entity-related specifications are included into the span. For this study, we use the long-span annotation layer as it fits best to the span characteristics of the other corpora.

##### Ex4CDS [35]

The dataset^7^ consists of short notes written by physicians in the context of estimating different patient risks. The text data has similarities to clinical text and was annotated with entities and relations and comprises entities such as “Condition”, “Lab Values”, “HealthState”, “Measure”, or “**Medication**”. The latter refers to, in this case, generic drug names, groups of medications, and active substances.

#### English dataset

##### CMED [36]

CMED^8^ was published by the organizers of the n2c2 challenge in 2022. It contains over 500 clinical notes based on the 2014 i2b2/ UTHealth Natural Language Processing shared task corpus [37–39] and is annotated with medication changes. Thus, every medication name is either labeled as “**Disposition**” (there was a change in medication), “**NoDisposition**” (no change in medication) and “**Undetermined**” (it is not evident from the context if there was a change or not)^9^. All “Disposition” events are further characterized with what kind of change happened (“Action”), e.g., if the medicated intake started, increased, or decreased, the timing of the change event (“Temporality”, e.g., past, present), the certainty of the medication change (“Certainty”, e.g., certain, hypothetical, etc.), the initiator of the change, i.e., the patient or physician (“Actor”) and if the medication event was negated or not (“Negation”).

#### French datasets

##### DEFT [40]

The DEFT corpus^10^ contains more than 700 documents from freely available clinical case reports in French and is a subset of the CAS corpus [41]. The data are classified into four general categories (“age”, “gender”, “outcome” and “origin”) and a subset of the reports is then annotated in a more fine-grained way, using, for instance, entity labels relating to physiology (e.g., “body measurement”) or surgeries (e.g., “surgical approach” or “medical device”). The entity we are interested in is the one named “**substance**”, a subset of the broader category of drug annotations, which also include labels like “concentration” or “mode”. “Substance” is defined as “commercial and generic drug names or generic substance” [40]. Note that not all documents in DEFT were annotated fully.

##### Quaero [42]

The Quaero French Medical Corpus^11^ was designed for medical NER and named entity normalization in Medline titles and EMEA documents. The types of medical entities follow the UMLS  [43] semantic groups and allow labels such as “Anatomy”, “**Chemical**”, or “Disorder”. Entities can be discontinuous and can be linked to more than one UMLS concept, the latter, however, is not relevant to the presented work. The label “Chemical” contains chemicals and drugs as defined by [44], including, for instance, antibiotics, clinical drugs, elements or enzymes, amongst others.

#### Spanish datasets

##### PharmaCoNER [45]

This corpus,^12^ developed for the PharmaCoNER shared task, contains approximately 1,000 manually annotated clinical case studies in Spanish. The annotated entities are “**Normalizables**” (chemicals^13^ that could be manually normalized to a CUI), “**No_Normalizables**” (chemicals that could not be normalized), “Proteinas” and “Unclear”.

##### CT-EBM-SP [46]

The Clinical Trials for Evidence-Based Medicine in Spanish corpus^14^ is annotated with entities from UMLS. The texts are taken, as the name suggests, from journal abstracts about clinical trials (500 documents) and announcements of trial protocols (700 documents), containing entities belonging to categories such as “Anatomy”, “Pathology”, or “**Chemical**”. The latter are defined as “pharmacological and chemical substances” [46].

In summary, we collected four German, one English, two French, and two Spanish datasets. All of these are based on similar, but not identical annotation guidelines and annotate entities that exhibit varying levels of semantic overlap with medication names. Note that although the guidelines might be comparable, the data were created with different goals in mind, by different annotators and in different settings. Therefore, the scope of the annotated entities might vary or include or exclude particular expressions.

### Pre-processing

All datasets are split into a training, development, and test set. In some cases (CMED, CT-EBM-SP, PharmaCoNER, Quaero), these splits were already given; the remaining corpora are split into 70% training, 15% development, and 15% test set. If possible, the data were split on document level, otherwise, e.g., in the case of BRONCO150, where several documents (sentences) were assigned to only five files, we take three files as training data and the remaining two as development and test sets. Code for data pre-processing, fine-tuning of models, and the error analysis is available online.^15^

First, all datasets are converted into the BRAT [47] format to prepare the input data by using modified scripts^16^ from the BRAT maintainers. This is done to re-use the existing evaluation script from n2c2 2022.^17^ We convert the data into CONLL-style  [48] inside-outside-beginning (IOB) format, which then serves as input to Huggingface’s  [49] transformer models. In the case of discontinuous entities, the longer span is selected over the single entities using the “longer span” option in the BRAT scripts. Due to the 512 token limit of our transformer model, we split the text into chunks of 26 sentences at maximum such that we can avoid issues of exceeding the token limit. To avoid the model getting biased towards one language during fine-tuning, we apply weighted random sampling when creating the batches fed to the model to make sure that each batch contains at least one example from each language. Note that when using this method, the model might see an example from a particular language and dataset several times during the fine-tuning process.

### Experimental setup

We rely on the base model of XLM-R [7] for all our experiments due to its lower computational costs, as we observed in preliminary trials that in terms of macro *F*_1_ score, the results showed only minor differences while fine-tuning with XLM-R *large* took significantly longer. Furthermore, the base XLM-R model works best for our use case compared to the other multi-lingual transformer models we tested. We fine-tune five models using five different seeds for every setup to account for training instabilities known from transformer models [5].

As outlined in the Section “Introduction”, we do not further address LLM-based medical NER within the context of our experiments. The broader reasoning is discussed in Section “Discussion”.

The predictions of the five resulting models on the test set are ensembled via a majority vote and evaluated against the gold standard data. After applying the fine-tuned models for inference, the resulting IOB sequences are converted back to BRAT format, allowing 1) some automated label corrections and 2) an easy and consistent evaluation using the n2c2 2022 evaluation script.^18^

#### Mono-lingual fine-tuning

In this setup, we fine-tune five XLM-R models on each language separately and evaluate the models on the complete test set bench of languages using the ensembled predictions. Hereby, the mono-lingual reference score as well as the cross-lingual transfer to other languages are tracked. We abbreviate these models as *mono*_*language*_, e.g., *mono*_*de*_.

#### Joint multi-lingual fine-tuning

The fine-tuning on all datasets across all languages constitutes the highest degree of joint multi-lingual training. We abbreviate this experiment setup to all. For this premise, we expect the model to learn a shared representation of medication names across languages by taking into account the different (language) contexts and annotation styles. While one can assume that this method may achieve lower scores due to the divergences across languages and datasets, the resulting model may be more robust in terms of dataset shifts, and it might be able to pick up, for instance, syntactic constructions that are evident in one dataset but not in another.

#### Fine-tuning on language pairs

The experiments of this group are based on the assumption that similar languages might learn from each other. Therefore, we combine the English and German training data (de+en, both are Germanic languages) into one dataset and the Spanish and French training data into one dataset (fr+es, both are Romance languages). The training data are selected to adequately counter imbalances in different scales of abundance of data samples within a language pair.

Merging the datasets from two linguistically related languages for a joint language-pair-specific training can be considered to constitute a middle ground between pure mono-lingual setups and the joint multi-lingual setup. These language-pair setups are relevant to investigate whether it is practical to stay within one language family instead of focusing on including also more remotely related languages.

## Results

Every model is evaluated on the same test set bench, containing examples of all languages and datasets (all) as well the language-specific subsets separately. To mitigate disagreement issues on exact span borders, we compute the scores for **P**recision, **R**ecall and $\mathbf{F_1}$ using the evaluation script from the n2c2 2022 challenge in overlap (lenient) mode. This ensures that also spans with (any) partial overlap are considered as true positive matches.^19^ The scores for exact matching can be found in the supplementary materials.

To provide a comprehensive yet condensed overview of our obtained results, the *F*_1_ scores on different experiment setups are given in Table 2.

**Table 2:**
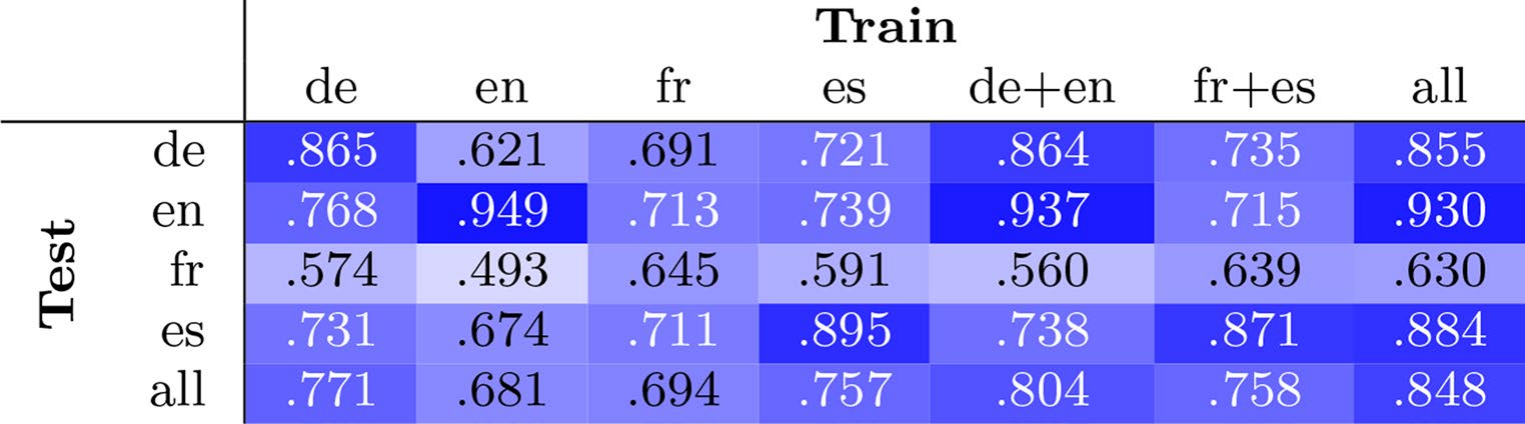
*F*_1_ scores observed for various mono-lingual, cross-lingual and multi-lingual setups. The scores are reported as micro scores over all test set samples and separated by language. “Train” denotes the data the model was fine-tuned on, “test” stands for the data the model was evaluated on

### Mono-lingual setups

With regard to the mono-lingual setup, three aspects are of particular interest:

- How well can a mono-lingual model learn to detect medication names within its own language?
- Which mono-lingual model performs well when it is required to transfer across all languages?
- Can mono-lingual models be used to transfer to certain other languages particularly well through the cross-lingual abilities of the XLM-R base model?

Addressing the first point, as for the mono-lingual models in general, we find, not surprisingly, that the best model for one language is always the one trained on this language. Regarding the second aspect, we observe that the model trained only on German data achieves the best lenient *F*_1_ (0.77) compared to the other “mono” models when evaluated on all examples, no matter the language. This is closely followed by the model trained only on Spanish data with an *F*_1_ of 0.76.

Further, it is interesting to see that the performance of model_en is lower compared to the other models when applied to the Spanish data ($F_1 = 0.67$ versus 0.73 (de) and 0.71 (fr)). The same is apparent for the French test set ($F_1 = 0.49$ versus 0.57 (de) and 0.59 (es)). According to the *F*_1_ scores, for French, training on Spanish is better than on German (if only by a small difference), while on Spanish, German is more beneficial than French. Note that French generally yields the lowest scores overall, even when trained mono-lingually. While the French language resources are composed of two datasets, the low results indicate conflicting annotation schemes.

Concerning the cross-lingual transfer of mono-lingually trained models, it appears that German and Spanish are best suited to be used for German, Spanish, or English cross-lingual transfer. While the German and Spanish language resources consist of more than one corpus per language, English only uses one dataset. This leads to the observed overfitting effect of the English mono-lingual model, which performs best within its own language domain at the expense of its cross-lingual scores on the German and Spanish test sets. In reverse, both German and Spanish mono-lingual models show rather evenly balanced scores in transfer setups.

### Joint multi-lingual setup

We discuss the results in the joint multi-lingual setup both on the language level and on the dataset level.

#### Language-level scores

We find that the results per language are slightly below those of the “mono-lingual” setups, but only by a small margin (de: 1% point, en: 1.9, fr: 1.5, es: 1.1). In short, this indicates that for our particular use case, there is no clear evidence that could prove a clear multi-lingual benefit over a mono-lingual when it comes to improving the scores for a certain target language.

#### Dataset-level scores

Going beyond the language-level analysis, we evaluate the joint multi-lingual model on each dataset independently. This indicates which datasets and implicitly which annotation guidelines are covered well by the jointly trained multi-lingual NER model. Table 3 displays these dataset-dependent scores.

**Table 3:** Dataset-dependent scores achieved by the joint multi-lingual model. The first part of the second column denotes the language (e.g., “de”), and the second part the dataset (e.g., “BRONCO150”). “train” denotes the data the model was fine-tuned on, “test” stands for the data the model was evaluated on. *p* = precision, *R* = recall

Train	Test Language	Test Corpus	P	R	$\mathbf{F_1}$
all	de	BRONCO150	0.845	0.888	0.866
all	de	Ex4CDS	0.714	0.294	0.417
all	de	GERNERMED	0.944	0.886	0.914
all	de	GGPONC	0.830	0.868	0.848
all	en	CMED	0.907	0.954	0.930
all	es	CT-EBM-SP	0.921	0.929	0.925
all	es	PharmaCoNER	0.755	0.885	0.815
all	fr	DEFT	0.186	0.568	0.281
all	fr	Quaero	0.889	0.599	0.716

The results of this multi-lingual model show two outliers: The *F*_1_ scores on the Ex4CDS dataset (0.41) and on the Deft corpus (0.28). In particular, a striking imbalance with regard to precision and recall scores on these outliers can be observed, as the German Ex4CDS shows much higher precision over recall, while it is reversed for the French DEFT dataset. We will go into more detail in the error analysis.

For the other datasets, the results appear to be quite good: The lowest *F*_1_ score apart from the two already mentioned is observed on the Quaero corpus, all others exceed an *F*_1_ of 0.81. In addition, the precision and recall values appear balanced.

### Language pair-based multi-lingual scores

When merging the languages by language families, Romance and Germanic, we can see several interesting results as well. First, the de+en cluster achieves the second best scores both on German and English, better than using the all model. The same holds true for fr+es model evaluated on French data. Further, the scores on Spanish are strongly reduced when using only de+en data, but when we compare the scores of de+en, fr+es and all, it seems like there is still some information gain from adding German and English examples to the French and Spanish data. This is evident in the improvement of the precision score by 2.4% points.

## Error analysis

The following is a mostly qualitative analysis of the predictions of the joint multi-lingual model. We highlight false positives (FPs) and false negatives (FNs) of interest and categorize them into groups for a more insightful overview. The counts of FP and FNs per language are reported in Table 4.

**Table 4:** Number of false positives (FP) and false negative (FN) samples. The individual samples are used for the qualitative error analysis

Language	#FP	#FN
German	376	382
English	113	63
French	298	175
Spanish	287	142
total	1,074	763
unique total	977	755

This might be an artifact of the different annotation guidelines of the various datasets: Since they were all built with different task objectives in mind, some drug occurrences might be annotated for some datasets but not for others, providing the model only with an unstable training signal on how to treat these occurrences.

### Analysis of false positives

FPs are text spans that were predicted as (part of) a drug name but are not correct according to the respective dataset’s ground truth annotation. During the qualitative analysis of the FP samples, we identify two notable error sources:

#### Annotation errors

Out of the collected false positive samples, several can be considered as *true* positives, contrary to the respective ground truth of the underlying dataset. For example, on the DEFT dataset the model predicted, among other things, “Rivotril” and “paroxétine”, both of which are, indeed, names of medications, yet they are not treated accordingly by the ground truth annotation.

Investigating the occurrences of the entities, we find that “Rivotril” only occurs in the Spanish training data and in no other dataset. “Paroxetine”, however, can be found in the training data of GGPONC and GERNERMED (“Paroxetin”), CMED (“Paroxetine”), PharmaCoNER and CT-EBM-S (“paroxetina”) and even in DEFT (“paroxétine”). Similar examples from German would be “Dopamin” (GGPONC) or “Metamizol” (BRONCO150, GGPONC), both were not labeled in the ground truth in some cases. However, we could verify them to be present in the training sets of GGPONC, PharmaCoNER, BRONCO150 and CT-EBM-SP. Consequently, we assume these to be annotation errors or entities that were not relevant for the respective corpus for some reason.^20^

#### Groups of other medical terms

In the FPs across all languages and datasets, we can find terms that belong to specific groups. These groups and their members often have medical associations, but are not medications themselves. However, their medical “context” might be a reason for their prediction. Some of the most visible groups are proteins (de: “Cyclin E”, en: “Creatine Kinase”, fr: “PHOSPHOMONOESTÉRASE”, es: “proteína C”), chemical compounds (de: “Dinitrotoluol”, en: “phosphate”, fr: “D-glycosylamines”, es: “fósforo”), abbreviations (de: “HLA”, en: “ASA”, fr: “STH”, es: “PTH”), general medication classes (de: “Medikation”, en: “pain medication”, es: “narcóticos”), medical terms and tools (de: “Gewebsflüssigkeit”, en: “Tegaderm”, fr: “solution”, es: “concentrado”), and dietary supplements (de: “Vitamin C”, en: “B12”, es: “calcio”). A reason for these predictions might be the label definitions of the different datasets. Some of them, e.g., Quaero and PharmaCoNER, include enzymes or chemical substances in their respective labels and the model is, apparently, not overfitting on any of those datasets. Also, the mentioned expressions are all used in very similar or even the same context as drugs, and therefore, the model might not be able to distinguish them semantically from medications.

Summarizing the analysis of FPs, we observe that most of the incorrectly detected expressions can be categorized into a particular group. Most of these classes can be associated with medicine, medical treatments or other things related to a clinical setting. Some FPs are simply based on annotation errors or on small differences in the dataset guidelines (e.g., “CHEM” versus “Medication”).

### Analysis of false negatives

Similar to the analysis of FPs, we now focus on entities that were classified as medication names according to their ground truth but were not detected by the multi-lingual model. During our analysis we identify the following group categories covering most FN entities:

- **therapies**: (de: “Sorafenibtherapie”, en: “lipid-lowering therapy”, fr: “traitement antidotique”)
- **abstract medication terms**: (de: “Herzentlastungsmedikamente”, en: “BP meds”, fr: “ANTICOAGULANTS”, es: “antitrombótica”)
- **brand names**: (de: “Sab Simplex”, fr: “IONSYS”, es: “McGhan”),
- **medications with imprecise spans**: (de: “Irinotecan (60 mg/m^2^”, fr: “Comprimé”)
- **ambiguous or weak terms**: (de: “B6”, en: “Mg”, fr: “CE”, es: “P”)

In contrast to that, we also encounter a few very long spans, e.g., “orale Supplemente mit Omega-3-Fettsäuren” (de, *oral supplements with omega-3 fatty acids*), “nouveau traitement antituberculeux” (fr, *new anti-tuberculosis treatment*), “antiveneno F(ab’)2 polivalente” (es, *polyvalent F(ab’)2 antivenom*). We will again refer to these long spans in the general observations below.

Finally, most FNs seem to be actual medication names (e.g., de: “Avelumab”, en: “LISINOPRIL”, fr: “Atripla”, es: “folato”) that were simply not detected by the model. The reason might be that some of these drugs (e.g., “Atripla”) were never seen in any training examples, or, in case they were seen, the context in the test example did not match the one the model was trained on.

### General observations

We conclude the qualitative error analysis with some general observations regarding the predicted entities.

#### Volatile span length

The model seems to have difficulties in deciding the span length of an entity. In terms of scores, this is ignored by the permissive overlap mode, but some true positives are conspicuously longer than they need to be from the perspective of annotating medication names. This might be due to the strikingly different span lengths across the training datasets: In GGPONC, PharmaCoNER, CT-EBM-SP, Quaero, and DEFT we have at least four medication names that are longer than four tokens, in the case of GGPONC there are 812 medications that are longer than four tokens. Also in GGPONC, DEFT and CT-EBM-SP we can still find several entities with a span longer than ten tokens. Examples from the German data are “fettlöslichen Vitaminen” (*fat-soluble vitamins*) or “orale Medikation” (*oral medication*). They were both predicted correctly, however, in other cases, e.g. “schwere Beruhigungsmittel” (*heavy sedatives*), this is not the case, since arguably a shorter span for “Beruhigungsmittel” (“sedative”) would have been correct.

#### Treatment versus medication

In several instances, there seems to be a disagreement between the terms of *treatment* (or other entity labels) and *medication*. Therapies, for example, like “chemo therapy” are dependent on the dataset and their respective annotation guidelines, categorized in either of these categories, and therefore predicted inconsistently.

#### Inconsistent annotations within datasets

We also encounter occurrences within datasets where the annotation might be misleading. For example, in one of the German datasets our system predicts both “Substanzen” (*substance*) and “Einzelsubstanzen” (*single substances*), but only the first of those is a correct match.

#### Overlap between false positives and false negatives

There are overall 59 expressions across all languages and datasets that are included in the FPs, but also in the FNs. Often, these belong to certain groups as specified above, e.g., general medication names (e.g., “medicación”), dietary supplements (e.g., “Magnesium”), or abbreviations (“ARV”). All of them, however, have a clear medical association. Their occurrence in both FPs and FNs may be a result of the different underlying guidelines or contexts, and there may be some annotation errors involved as well. However, it also demonstrates the difficulty of annotating medical texts and creating guidelines for the annotation.

#### Unseen medications

To make sure the model is not simply overfitting to individual medication names, we check for some true positives if they occur in any of the training sets. Indeed, we observe that there are several correctly predicted drugs that the model did not see during training. Examples are “Quixidar” (Quaero), and “rifampine” (DEFT). “Dexamethasone” is an interesting case: We can see that it was correctly predicted in both GERNERMED and GGPONC, but it never occurred like that in the training data. Instead, it was included in much longer spans, e.g. “für 3 Tage 5 mg Dexamethasone” (*for 3 days 5 mg Dexamethasone*). Finally, examples for Spanish are “biperideno” (PharmaCoNER) or “tirofibán” (CT-EBM-SP). From this, we can conclude that the context indeed plays a role when detecting medication names.

## Discussion

In our experiments, we show that the multi-lingual model achieves a *F*_1_ score that is only slightly below the one of the “mono-lingual” models when evaluated on the languages separately. Since the difference is indeed very small (the maximum difference in lenient *F*_1_ score is 1.9% points), this can be good news for certain use cases: Given the case when several languages need to be processed, a multi-lingual model needs less training time and computational resources, may be more robust to dataset shifts and potential noise like spelling errors, and can be easier to use in practice.

As part of the initial research question, the findings on the cross-lingual transfer indicate that transfer across certain languages is in fact a viable solution. While we observed drops in performance, our findings indicate that conflicting annotation guidelines across different datasets might be a larger impeding factor than lossy effects during the cross-lingual transfer. For instance, the transfer of a mono-lingual French model to English yielded quite good scores already.

Concerning language pairs, the results vary. Assuming there is no target language training material available, combining other languages for fine-tuning does indeed show good performance on the target language and also often performs better than fine-tuning only on one source language. Since this is not always the case, a thorough inspection of the available data might be necessary, to avoid the introduction of noise. For language pairs, languages from the same family seem to work better.

On two datasets, Ex4CDS (de) and DEFT (fr), we find a lower performance when compared to the other corpora (0.41 and 0.28 *F*_1_). This might be due to the smaller dataset size (Ex4CDS), the different guidelines for both corpora (e.g., Ex4CDS contains, in contrast to the other datasets, explanations of clinical decisions), and the annotation of DEFT, where some documents were not completely annotated. Nevertheless, we use this dataset to investigate the performance of the system in low-resource contexts, and find, not surprisingly, a high number of false positives according to the evaluation scores. Many of these false positives, however, are actually correctly identified medication spans when taking a closer look.

With respect to both false positives and false negatives, we find error groups that are evident across all languages and across all datasets. We *cannot* say that there are language-specific errors made by the system. It is, therefore, not the case that the model overfit on one language or dataset, most mistakes are to be found cross-lingually. For future work, it would be interesting to take a closer look at the contexts of the predicted FPs and FNs that we cannot explain by their medical association, context, or inconsistencies in the annotation.

We observe overlaps between false positives and false negatives in all languages and datasets. This hints at annotation inconsistencies, but also on very subtle differences that might depend on the exact context in which the entity in question was uttered. We argue that this is a normal phenomenon of manually annotated datasets, especially in a more complex domain like the medical domain.

Since all the datasets used in this work are based on different annotation guidelines, it is no surprise that for some of the test sets, we find predictions that are evaluated as false positives. These might be correct for one dataset, but not for the other. However, this shows very clearly why it is important to take a look at the actual predictions and not only at the scores: If we would like to (semi-)automatically annotate a new medical and potentially multi-lingual dataset, these predictions would still be very useful. Also, as we have seen in some examples, even if a particular drug was not present in the training data, it can still be predicted correctly, based on context, but also based on its potential occurrence in the other datasets.

Finally, the fact that a lot of medication names are very similar across the four investigated languages (e.g., compare “Paroxetin” vs. “Paroxetine” vs. “paroxétine” vs. “paroxetina”) is likely to have a positive impact on the drug detection task as well. This might change for drug names with different origins or when using datasets from other language families, and maybe more importantly, other scripts. However, inconsistencies within datasets and in label definitions across datasets might counterbalance these effects. The investigation of the influence of inconsistencies is, however, a task for future work.

Regarding the use of LLMs for medical NER tasks, these models have demonstrated applicability in areas such as medication detection  [25]. However, a direct and fair comparison of their cross-lingual capabilities with masked language models is inherently challenging due to fundamental architectural and operational differences between these model types. While our study focuses on gradient-based fine-tuning for the medication detection task, LLMs are typically employed using few-shot prompting techniques to circumvent the computational expense of fine-tuning larger model sizes. These conceptual differences not only affect performance but also introduce distinct categories of error, which require separate analytical frameworks. Therefore, our study remains centered on a single model, XLM-R, to ensure a focused and consistent evaluation.

## Conclusions

In this work, we investigated the ability of the cross- and multi-lingual transfer-learning capabilities of the XLM-R model in the context of medication detection in different languages and datasets. We fine-tuned the model on mono-lingual, bi-lingual and multi-lingual datasets and evaluated their drug detection performance across all languages. While our results indicate that mono-lingual models perform best on their respective target language, multi-lingual-trained models can reach scores close to their mono-lingual counterparts. Due to their cross-lingual transfer, we demonstrated that multi-lingual models can be a relevant approach in low-resource contexts in order to tackle NLP tasks with non-native datasets even if no appropriate native dataset or no language-specific pre-trained model is available.

An error analysis provided valuable insights into the mistakes the multi-lingual model makes when extracting medication names from unseen data. The found error groups allow further investigations into how these errors can be alleviated or even avoided, e.g., by more consistent annotation guidelines across languages. This stresses the need to strengthen the efforts towards more standardized, comparable and interoperable annotation guidelines in general. We also find indications that the model learns across dataset boundaries, taking into account drug names that were only present in another language’s dataset.

More medical datasets and annotation data for extended evaluation of multi-lingual models could further improve the state of medical NLP in low-resource contexts, yet due to our scope this is considered future work. Furthermore, the usefulness of multi-lingual models in other language families (e.g., Arabic, Swedish, Ukrainian or Japanese) for the identical clinical purpose of drug detection remains open for further investigation.

## Electronic supplementary material

Below is the link to the electronic supplementary material.

Supplementary Material 1: Model Parameters

Supplementary Material 2: Dataset Statistics

Supplementary Material 3: Verbose Scores

## Data Availability

All relevant code is available at the GitHub repository at https://github.com/lraithel/cross_ling_drug_ner. The referenced datasets may be requested from their respective authors.

## Footnotes

1 https://n2c2.dbmi.hms.harvard.edu/2022-amia-workshop

2 https://www2.informatik.hu-berlin.de/%7Eleser/bronco/index.html

3 http://www.dimdi.de/dynamic/de/arzneimittel/atc-klassifikation/

4 https://github.com/frankkramer-lab/GERNERMED

5 https://www.leitlinienprogramm-onkologie.de/projekte/ggponc-english/

6 Annotation guidelines of GGPONC: https://github.com/hpi-dhc/ggponc_annotation/blob/master/annotation_guide/anno_guide.pdf

7 https://github.com/DFKI-NLP/Ex4CDS

8 To the best of our knowledge, these data are not (yet) publicly accessible.

9 Note that for the CMED dataset, all medication labels in the test set are already mapped to “Drug”. Unfortunately, we do not know the exact definition of “Drug”.

10 https://deft.limsi.fr/2019/index-en.html

11 https://quaerofrenchmed.limsi.fr/

12 https://temu.bsc.es/pharmaconer/

13 For this dataset, the terms “chemical” and “drug” are used interchangeably.

14 http://www.lllf.uam.es/ESP/nlpmedterm_en

15 https://github.com/lraithel/cross_ling_drug_ner

16 brat2bio: https://github.com/spyysalo/standoff2conll and bio2brat https://github.com/nlplab/brat/blob/master/tools/BIOtoStandoff.py

17 https://github.com/lraithel/cross_ling_drug_ner/blob/main/src/eval_script.py

18 https://n2c2.dbmi.hms.harvard.edu/2022-track-1

19 Note that if there is nothing to annotate/predict in a document, the evaluation script returns 0.0 for all scores.

20 Note that some of the DEFT examples were only annotated partially.

## Abbreviations

NLP: Natural language processing
NER: Named entity recognition
LLM: Large language model
ADR: Adverse drug reactions
IOB: Inside-outside-beginning
FP: False positive
FN: False negative
