# Supplementary material for: Cross- & multi-lingual medication detection: a transformer-based analysis
Source: BMC Med Inform Decis Mak. 2025 Oct 2;25:359. doi: 10.1186/s12911-025-03179-1 (PMC12490045; doi:10.1186/s12911-025-03179-1)
Supplement: Supplementary file 1 — Supplementary Material 1: Model Parameters [file 12911_2025_3179_MOESM1_ESM.pdf]

# Model Parameters

All models were trained using batches of size 8, 5,000 warm-up steps, and a weight decay of 0.002. The seeds we used for the different runs were 42, 712, 9721, 26747, and 424881. Hyper-parameters were determined using the Weights & Biases framework[1] framework and optimizing for $F_1$ score. Each chunk of data contained a maximum of 26 sentences. The sentence split was done using the original BRAT scripts as described in the pre-processing section. The learning rates are provided in Table 1.

| **Model** | **Learning Rate** |
|---|---|
| mono_de | 9.98e-6 |
| mono_en | 9.98e-5 |
| mono_fr | 9.98e-5 |
| mono_es | 9.98e-5 |
| all | 9.98e-6 |
| de_en | 9.98e-6 |
| fr_es | 9.98e-6 |
| original labels | 9.98e-6 |

Table 1: The learning rates of the different models

[1] `https://wandb.ai/`
