# Supplementary material for: Cross- & multi-lingual medication detection: a transformer-based analysis
Source: BMC Med Inform Decis Mak. 2025 Oct 2;25:359. doi: 10.1186/s12911-025-03179-1 (PMC12490045; doi:10.1186/s12911-025-03179-1)
Supplement: Supplementary file 2 — Supplementary Material 2: Dataset Statistics [file 12911_2025_3179_MOESM2_ESM.pdf]

Dataset Statistics

The statistics on the drug-related labels are shown in Table 1.

	Dataset	# Labels (Drug)	ø$\frac{\#tokens}{label}$ (Drug)	ø$\frac{\#chars}{label}$ (Drug)
de	BRONCO150	1,630	1.16	10.07
	GERNERMED	1,450	1.22	10.76
	GGPONC 2.0	23,671	1.73	14.64
	Ex4CDS 2.0	98	1.11	7.32
en	CMED	8,993	1.15	8.44
fr	Quaero	3,537	1.29	10.86
	DEFT	1,337	1.44	12.97
es	PharmaCoNER	4,448	1.15	10.04
	CT-EBM-SP	9,224	1.34	11.53
Total		49,128	1.48	12.57

Table 1: Dataset Statistics only on drug-related labels. Tokenization performed using SpaCy.
