# Supplementary material for: Cross- & multi-lingual medication detection: a transformer-based analysis
Source: BMC Med Inform Decis Mak. 2025 Oct 2;25:359. doi: 10.1186/s12911-025-03179-1 (PMC12490045; doi:10.1186/s12911-025-03179-1)
Supplement: Supplementary file 3 — Supplementary Material 3: Verbose Scores [file 12911_2025_3179_MOESM3_ESM.pdf]

Results including Strict and Lenient Scores

The resulting scores of the model trained in the multilingual setting are shown in Table 1, including the scores with strict span-matching.

		Strict			Lenient		
**train**	**test**	**P**	**R**	**F_1**	**P**	**R**	**F_1**
all	all	.731	.750	.740	.838	.860	.848
	de	.642	.662	.652	.842	.869	.855
	en	.877	.922	.899	.907	.954	.930
	fr	.589	.527	.556	.667	.596	.630
	es	.824	.879	.851	.857	.914	.884

Table 1: Results of the multi-lingual model trained and evaluated on all languages.

The scores of the same multilingual model evaluated on the individual corpora are given in Table 2, for both lenient, and strict span-matching settings.

		Strict			Lenient		
**Train**	**Test**	**P**	**R**	**F_1**	**P**	**R**	**F_1**
all	de_BRONCO150	.793	.834	.813	.845	.888	.866
all	de_Ex4CDS	.429	.177	.250	.714	.294	.417
all	de_GERNERMED	.859	.806	.832	.944	.886	.914
all	de_GGPONC	.602	.630	.616	.830	.868	.848
all	en_CMED	.877	.922	.899	.907	.954	.930
all	es_CT-EBM-SP	.883	.890	.887	.921	.929	.925
all	es_PharmaCoNER	.731	.858	.789	.755	.885	.815
all	fr_DEFT	.163	.496	.245	.186	.568	.281
all	fr_Quaero	.786	.530	.633	.889	.599	.716

Table 2: Results separated by dataset. The model was trained on all languages (`all`). The first part of the second column denotes the language (e.g. "de"), the second part the dataset (e.g. "BRONCO150").

The evaluation of different models, as provided in Table **??** in the main manuscript, are shown in Table 3 with their additional precision and recall values with lenient span matching.

The results of the mono-lingual models evaluated on various languages are shown in Table 4 in both strict and lenient span matching settings.

The results of the models trained without label harmonization are shown in Table 5 for various settings.

The results of the models trained on pair-wise language clusters are shown in Table 6 for both strict and lenient span matching configurations.

**Train**	**Test**	**P**	**R**	$\mathbf{F_1}$
	all	.838	.860	**.848**
	de	.842	.869	.855
all	en	.907	.954	.930
	fr	.667	.596	.630
	es	.857	.914	.884
	all	.817	.791	.804
	de	.863	.866	.864
de+en	en	.926	.948	.937
	fr	.603	.522	.560
	es	.766	.712	.738
	all	.743	.773	.758
	de	.742	.728	.735
fr+es	en	.667	.771	.715
	fr	.653	.626	.639
	es	.833	.913	.871
	all	.733	.813	.771
	de	.856	.874	**.865**
de	en	.687	.870	.768
	fr	.573	.575	.574
	es	.673	.801	.731
	all	.740	.630	.681
	de	.646	.598	.621
en	en	.963	.934	**.949**
	fr	.610	.414	.493
	es	.785	.590	.674
	all	.752	.644	.694
	de	.756	.635	.691
fr	en	.752	.678	.713
	fr	.671	.622	**.645**
	es	.791	.645	.711
	all	.792	.725	.757
	de	.757	.688	.721
es	en	.804	.684	.739
	fr	.632	.554	.591
	es	.901	.889	**.895**

Table 3: All scores of models trained in the mono-lingual and multi-lingual settings. The scores are reported as micro scores over all test set samples and separated by language. Best scores are marked in bold font. The best score when training on one language and evaluating on all languages is underlined. “Train” denotes the data the model was fine-tuned on, “Test” stands for the data the model was evaluated on. P = precision, R = recall.

		Strict			Lenient		
**Train**	**Test**	**P**	**R**	$\mathbf{F_1}$	**P**	**R**	$\mathbf{F_1}$
de	all	.589	.653	.619	.733	.813	**.771**
	de	.656	.670	.663	.856	.874	**.865**
	en	.616	.780	.688	.687	.870	.768
	fr	.479	.481	.480	.573	.575	.574
	es	.529	.630	.575	.673	.801	.731
en	all	.617	.525	.567	.740	.630	.681
	de	.449	.415	.431	.646	.598	.621
	en	.934	.906	.920	.963	.934	**.949**
	fr	.529	.359	.428	.610	.414	.493
	es	.704	.529	.604	.785	.590	.674
fr	all	.598	.512	.552	.752	.644	.694
	de	.490	.412	.447	.756	.635	.691
	en	.688	.620	.652	.752	.678	.713
	fr	.589	.547	.567	.671	.622	**.645**
	es	.707	.576	.635	.791	.645	.711
es	all	.657	.602	.628	.792	.725	.757
	de	.508	.461	.483	.757	.688	.721
	en	.740	.629	.680	.804	.684	.739
	fr	.544	.476	.508	.632	.554	.591
	es	.865	.853	.859	.901	.889	**.895**

Table 4: Results of models trained on the single languages. The evaluation scores are reported as micro scores over all test set samples and separated by language. Best scores are marked in bold font. The best score when training on one language and evaluating on all languages is underlined. de = German, en = English, fr = French, es = Spanish. P = precision, R = recall.

Label Name	Train	Test	Strict			Lenient		
			**P**	**R**	**F_1**	**P**	**R**	**F_1**
Substance	all	all	.607	.591	.599	.824	.802	.813
Medication			.000	.000	.000	.167	.059	.087
MEDICATION			1.00	.003	.006	1.000	.003	.006
substance			.174	.200	.186	.188	.216	.201
CHEM			.698	.727	.712	.747	.779	.763
Drug			.599	.554	.575	.642	.594	.617
NO_NORMALIZABLES			.000	.000	.000	.000	.000	.000
NORMALIZABLES			.888	.668	.763	.915	.689	.786
MEDICATION	all	de_BRONCO150	1.00	.003	.006	1.000	.003	.006
Medication		de_Ex4CDS	.000	.000	.000	1.000	.059	.111
Drug		de_GERNERMED	.885	.813	.847	.940	.863	.900
Substance		de_GGPONC	.621	.591	.606	.843	.802	.822
Drug	all	en_CMED	.869	.487	.624	.936	.524	.672
CHEM	all	es_CT-EBM-SP	.887	.879	.883	.930	.922	.926
NO_NORMALIZABLES		es_PharmaCoNER	.000	.000	.000	.000	.000	.000
NORMALIZABLES		es_PharmaCoNER	.919	.668	.774	.948	.689	.798
substance	all	fr_DEFT	.180	.200	.189	.194	.216	.205
CHEM		fr_Quaero	.785	.504	.614	.886	.568	.692

Table 5: Results of the model trained with the original labels. The label names are listed in the left-most columns. We only report scores for the respective dataset labels. Note that the reported scores only reflect if the correct entity *and* correct label were found.

Train	Test	Strict			Lenient		
		**P**	**R**	**F_1**	**P**	**R**	**F_1**
de_en	all	.685	.663	.674	.817	.791	.804
fr_es	all	.615	.639	.627	.743	.773	.758
de_en	de	.663	.666	.665	.863	.866	**.864**
de_en	en	.895	.916	.905	.926	.948	**.937**
de_en	fr	.518	.449	.481	.603	.522	.560
de_en	es	.650	.604	.626	.766	.712	.738
fr_es	de	.498	.488	.493	.742	.728	.735
fr_es	en	.603	.697	.647	.667	.771	.715
fr_es	fr	.578	.553	.565	.653	.626	**.639**
fr_es	es	.794	.870	.830	.833	.913	.871

Table 6: The results of the cluster approaches.
